# Water Arsenic Exposure and Children’s Intellectual Function in Araihazar, Bangladesh

**DOI:** 10.1289/ehp.6964

**Published:** 2004-04-28

**Authors:** Gail A. Wasserman, Xinhua Liu, Faruque Parvez, Habibul Ahsan, Pam Factor-Litvak, Alexander van Geen, Vesna Slavkovich, Nancy J. Lolacono, Zhongqi Cheng, Iftikhar Hussain, Hassina Momotaj, Joseph H. Graziano

**Affiliations:** ^1^Department of Psychiatry, College of Physicians and Surgeons, Columbia University, New York, New York, USA; ^2^New York State Psychiatric Institute, New York, New York, USA; ^3^Mailman School of Public Health and; ^4^Lamont-Doherty Earth Observatory, Columbia University, New York, New York, USA; ^5^National Institute of Preventive and Social Medicine, Dhaka, Bangladesh; ^6^Columbia University Bangladesh Arsenic Project, New York, New York, USA

**Keywords:** arsenic, children, IQ

## Abstract

Exposure to arsenic has long been known to have neurologic consequences in adults, but to date there are no well-controlled studies in children. We report results of a cross-sectional investigation of intellectual function in 201 children 10 years of age whose parents participate in our ongoing prospective cohort study examining health effects of As exposure in 12,000 residents of Araihazar, Bangladesh. Water As and manganese concentrations of tube wells at each child’s home were obtained by surveying all wells in the study region. Children and mothers came to our field clinic, where children received a medical examination in which weight, height, and head circumference were measured. Children’s intellectual function on tests drawn from the Wechsler Intelligence Scale for Children, version III, was assessed by summing weighted items across domains to create Verbal, Performance, and Full-Scale raw scores. Children provided urine specimens for measuring urinary As and creatinine and were asked to provide blood samples for measuring blood lead and hemoglobin concentrations. Exposure to As from drinking water was associated with reduced intellectual function after adjustment for sociodemographic covariates and water Mn. Water As was associated with reduced intellectual function, in a dose–response manner, such that children with water As levels > 50 μg/L achieved significantly lower Performance and Full-Scale scores than did children with water As levels < 5.5 μg/L. The association was generally stronger for well-water As than for urinary As.

In Bangladesh, approximately 30–40 million people (British Geological Survey 2001) have been chronically exposed to high concentrations of naturally occurring arsenic in drinking water, supplied by approximately 10 million tube wells (British Geological Survey 2001; van Geen et al. 2002). Aside from carcinogenic and vascular effects, the literature contains reports (e.g., Rodriguez et al. 2003) of neurologic consequences of acute and chronic exposure in adults, although the dosimetry is poorly described. Clinical and industrial reports of heavy exposure in adults (Bolla-Wilson and Bleecker 1987; Morton and Caron 2003) document adverse impacts on a range of cognitive functions, including learning, memory, and concentration, as well as peripheral and central neuropathies (Morton and Dunnette 1994; Pershagen et al. 1981; Schoolmeester and White 1980).

In addition to elevated As concentrations in Bangladesh groundwater, the British Geological Survey (2001) has reported that many of the existing wells in Bangladesh also have manganese concentrations that exceed the World Health Organization (WHO) standard of 500 μg/L. Occupational Mn exposure has been associated with neurologic sequelae in adults, specifically Parkinsonism (Gorell et al. 1999).

Given the absence of a significant research base concerning the consequences of As or Mn in children, we sought to examine the possible associations between As exposure and intellectual function, taking into account possible effects of Mn. In 2000, we began a prospective study of the health effects of As in 12,000 adult residents of Araihazar, Bangladesh. The study site, a 25-km^2^ region located approximately 30 km east of Dhaka, was chosen because of its wide range of As concentrations in drinking water. Our survey of 6,000 contiguous wells in the region (van Geen et al. 2003b) revealed that 75% exceed the WHO As standard of 10 μg/L and 53% exceed the Bangladesh standard of 50 μg/L; water As concentrations ranged from < 1 to 900 μg/L. In an analysis of a subset of 629 well-water samples, 82% exceeded the WHO Mn standard (Cheng et al. 2004).

In the same region, we are examining the consequences of As exposure on children’s health. We report here the results of a cross-sectional investigation of intellectual function in 201 children 10 years of age.

## Materials and Methods

### Overview.

The present project is part of a larger ongoing multidisciplinary study by health, earth, and social scientists working collaboratively in Araihazar, Bangladesh. People in Araihazar, as in most of rural Bangladesh, live in houses with cement or mud floors and tin or straw walls and roofs. Members of extended families live in clusters of individual houses (called a *bari*), surrounded by family farmland. Each bari has one or more tube wells, usually owned by a senior family member. This region is not particularly poor by Bangladesh standards.

Given the absence of a research base concerning the effects of As on children’s intellectual functioning, we extrapolated our assessment plan from experience gained in 12 years of studying lead exposure in Kosovo, Yugoslavia (e.g., Factor-Litvak et al. 1999). That research, consistent with the findings of others, documented adverse impact of Pb exposure on intellectual development, with effects stronger on visuomotor than on verbal functioning.

### Subjects.

Of the 11,749 adults enrolled in our cohort study, we selected at random a pool of 400 of their children (using 400 different wells) between 9.5 and 10.5 years of age. During summer of 2002, field staff visited families at home to verify child age and school attendance, to discuss the proposed study, and to make an appointment for a clinic visit. Of the initial pool of 400, 176 children were assessed during the summer of 2002. Informed parental consent and assent of the children were obtained. The study was approved by the Bangladesh Medical Research Council and the Columbia University institutional review board. Of the remaining 224 potential study children, 105 were never visited because seasonal flooding made access hazardous; of those visited, 14 families refused participation and 8 were excluded because the child was not attending school or was > 10.5 years of age (*n* = 33), the family was not at home, or other, unspecified reasons (*n* = 64). To bolster our sample size, in 25 instances when a home visit identified an excluded child, interviewers selected a child of participating parents from the same village until 201 children, using 196 wells, had been assessed.

### Procedure.

Children and their mothers came to our field clinic, where children participated in the assessments described below and received a medical examination by a study physician. Weight, height, and head circumference were measured. Children provided urine specimens for the measurement of urinary As and creatinine and were asked to provide a blood sample for the measurement of blood lead (BPb) and hemoglobin (Hgb) concentrations. Of the 201 children assessed, 107 agreed to provide blood samples. Information on family demographics (e.g., parental education, occupation, housing type) was available from the baseline interview of parents during their enrollment in the cohort study. Information on the primary source of drinking water was obtained from the child’s mother. Parents were asked whether their home included a television; about parental age, education, and occupation; and about child birth order. For an additional surrogate for social class, the type of roofing on the well owner’s home was recorded as thatched, tin, or cement (thatched lowest, cement highest). Children were given a toy as thanks for their participation; families participating in the larger cohort study receive primary medical care at our own field clinic.

### Measures.

#### Water analyses.

Water As concentrations of tube wells at each child’s home were obtained during a survey of all wells in the study region (van Geen et al. 2003b) and shipped to Columbia University’s Lamont Doherty Earth Observatory for analysis. Water samples were analyzed by graphite furnace atomic absorption (GFAA), which had a detection limit of 5 μg/L. Those water samples found to have < 5 μg/L were subsequently reanalyzed by inductively coupled plasma–mass spectrometry (ICP-MS), which has a detection limit of 0.1 μg/L (Cheng et al. 2004). Of the 196 well-water samples, 194 were also analyzed for Mn by standard flame atomic absorption spectrophotometry.

#### Biochemical measurements.

Urinary As concentrations were assayed by GFAA at the Mailman School of Public Health, using a Perkin-Elmer Analyst 600 system as previously described (Nixon et al. 1991). Our laboratory participates in a quality control program coordinated by P. Weber at the Québec Toxicology Center (Québec City, Québec, Canada). During the course of this study, intraclass correlation coefficients between our laboratory’s values and samples calibrated at Weber’s laboratory were 0.99. Levels of As in urine were also adjusted for urinary creatinine levels, which were analyzed by a colorimetric Sigma Diagnostics Kit (Sigma, St. Louis, MO, USA). In addition, urinary As metabolites were speciated using a method adapted after Heitkemper et al. (2001). This method employs high-performance liquid chromatography separation of arsenobetaine (AsB), arsenocholine (AsC), arsenate, arsenite, monomethylarsonic acid (MMA), and dimethylarsinic acid (DMA), followed by detection by ICP-MS. The percentages of inorganic As (InAs; i.e., arsenate + arsenite), MMA, and DMA were calculated after subtracting AsC and AsB from total urinary As.

Venous blood samples were obtained for measurements of BPb (Fernandez and Hilligoss 1982) and Hgb. Whole-blood samples were appropriately stored and transported to a laboratory at Columbia University that participates in the BPb quality control program of the Centers for Disease Control and Prevention (CDC; Atlanta, GA, USA). Intraclass correlation coefficients between our laboratory’s values and samples calibrated at CDC ranged between 0.97 and 0.99. Children providing blood samples had mothers with significantly more years of education and received significantly higher Verbal raw scores than did mothers of children not providing blood samples (Wilcoxon tests, degrees of freedom = 1, *p*-values < 0.05); there were no other differences between those providing and not providing blood samples.

#### Children’s intellectual function.

The Wechsler Intelligence Scale for Children, version III (WISC-III; Wechsler 1991), suitable for children ≥ 6 years of age, consists of five (or six, depending upon administration) verbal subtests, which together provide a Verbal intellectual quotient (IQ) score, and a similar number of performance subtests that together provide a Performance IQ. Neither the WISC-III (Wechsler 1991) nor any other recently well-standardized child IQ test has been adapted or standardized for use in Bangladesh.

In Araihazar, living conditions differ dramatically from those in Western settings where this test was developed, which necessitated adaptations for use in this culture. For example, a typical home consists of a single room, often with a dirt floor. Most families use biomass fuel (leaves, hay, dung) for cooking. Electricity is available in most homes; commonly this consists of one or two bulbs used for lighting. Many common Western household items, such as telephones and bathtubs, are rare.

We used six subtests that seemed the most culturally adaptable to this cultural context. Of the WISC-III Verbal subtests, we used Similarities and Digit Span: Of the Performance subtests, we used Picture Completion, Coding, Block Design, and Mazes. Two items with no recognizable analog were eliminated from the Picture Completion subscale (telephone, bathtub), and close substitutions were made for four others from the Similarities subscale (“mango and banana” for “apple and banana”; “flute and drum” for “piano and guitar”; “dog and cow” for “cat and mouse”; and “tire and ball” for “wheel and ball”). The WISC-III subtests include items of graduated difficulty, with more points awarded for harder items or faster completion. We summed these weighted items across Verbal, Performance, and Full-Scale domains to create Verbal, Performance, and Full-Scale raw scores; we also transformed these into measures of estimated Verbal, Performance, and Full-Scale IQ, using procedures presented in the test manual (Wechsler 1991), despite the obvious limitations in application to this population. Below we use “IQ” to represent this estimated measure.

Maternal intelligence was assessed with Raven’s Standard Progressive Matrices, a non-verbal test relatively free of cultural influences (Raven et al. 1983).

### Translation and training.

All tests and interviews were translated (and back-translated) between Bangla (Bengali) and English. As noted, items deemed to be culturally inappropriate were altered or omitted. Materials were piloted to ensure maternal and child comprehension; two interviewers were then trained by a competent tester (G.A.W.) and then continued with supervised practice sessions for 2 weeks. All written test responses were rechecked when data were sent to the Columbia University Department of Psychiatry for entry.

### Statistical analyses.

#### Outcomes.

Because of concerns about the application of U.S. standardization of the WISC-III to Bangladeshi children, we first conducted analyses that predicted Verbal, Performance, and Full-Scale raw scores. Because the psychometric properties of IQ scores are more familiar to readers, we also applied the same analytic models to the prediction of estimated Verbal “IQ,” Performance “IQ,” and Full-Scale “IQ.”

#### Covariate adjustment.

In Bangladesh, grammar school extends to fifth grade. Therefore, mother’s and father’s education were categorized as none, 1–5 years, and 6–13 years. Parental occupation was recoded as laborer/farmer, factory/other paid job, business, or missing/other. Because just 6% (*n* = 11) of mothers reported working outside the home, only paternal occupation was included in the regression models. From a pool of potential demographic covariates, we retained those that were empirically or theoretically importantly related to child intelligence, as well as those that made an initial contribution (at significance *p* ≤ 0.20 or better), in initial regression analyses, either to any of the outcomes of interest or to the measures of As exposure.

#### Analytic model.

The analyses first sought to predict the outcomes of interest from the set of sociodemographic factors using linear regression models; once this “core” model was derived, we examined the incremental association of exposures (Mn, As) singly and together, measured continuously. We repeated our analyses, categorizing children into groups, based on quartiles of water As to illustrate dose–response relationships. We next repeated these analyses for the subset of children providing blood samples for the measurement of BPb and Hgb, measured continuously. In all analyses, BPb and water As were log-transformed and water Mn was square root–transformed to make distributions approximately symmetric.

For the most part, analyses are based on *n* = 201 children. Analyses considering well-water Mn employ *n* = 194. Analyses involving urinary As and its metabolites are based on *n* = 200; for analyses considering BPb and Hgb, *n* = 107.

## Results

### Sample characteristics.

Table 1 presents descriptive information for all demographic, water, and biochemical variables. Average child age was 10 years; approximately half the children in the sample were male; one-third had regular access to a television. On average, mothers and fathers reported 3.7 and 2.9 years of education, respectively. Average child height was 125.6 cm, and average weight was 21.9 kg, values that correspond to roughly the fourth percentile by U.S. norms (CDC 2003).

### Exposure characteristics.

Water As concentrations ranged from 0.094 to 790 μg/L, with a mean (117.8 μg/dL) and distribution comparable to those in the larger set of 6,000 contiguous wells in Araihazar (van Geen et al. 2003b). The mean water Mn concentration of 1,386 μg/L was well in excess of the U.S. and WHO recommended maximum contaminant level (MCL) of 500 μg/L, with a range up to 5,438; water Mn values are not available for the entire set of 6,000 wells. Indeed, 82% of children were consuming water in excess of the MCL for Mn. The association between water As and water Mn was significant (Spearman *r* = 0.39; *p* < 0.0001) but not strong enough to preclude examination of their independent effects on child intelligence. The correlation between water As and urinary As (Spearman *r* = 0.45, *p* < 0.0001) was comparable to that previously reported for adults in this region (Ahsan et al. 2000). In the subsample of children for whom BPb measures were obtained (*n* = 107), Spearman correlations (necessitated by skewed distributions) with well-water As (−0.16 ) and with urinary As (−0.06) were not significant.

### Relationship between covariates and intellectual function.

Linear regression analyses predicting test raw scores from the sociodemographic features retained in the final “core” model revealed generally better scores in children of more educated mothers and of mothers with higher Raven scores, those living in more adequate dwellings, those with access to television, and those who were taller and had larger head circumference (data not shown).

### Relationship between well-water Mn and intellectual function.

Without adjustment for either core variables or water As, water Mn was significantly associated with Full-Scale and Performance raw scores (*B*-values = −0.33 and −0.29, *p* < 0.002 and *p* < 0.001, respectively) but not with Verbal raw score (*B* = −0.04, *p* = 0.15). Addition of Mn to analytic models made little change in associations between core model variables and intellectual function raw scores. Controlling for sociodemographic features, Mn levels were significantly negatively associated with Performance and Full-Scale raw scores (*B*-values = −0.20 and −0.22, respectively, *p* < 0.03) but not with Verbal raw score (*B* = −0.02, *p* > 0.5). However, when water As was added to these models, Mn made no significant (*p* > 0.25) contribution to intellectual function. With both water As and water Mn in the model, there was no significant interaction in their prediction of Full-Scale, Verbal, or Performance raw scores.

### Relationship between well-water As and intellectual function.

Table 2 presents associations between water As and intellectual function, before and after adjustment for sociodemographic features. In each case, associations between water As and intellectual function raw scores were stronger before adjustment for sociodemographic features. In unadjusted analyses, water As explained 7.29, 2.61, and 7.04% of the variance in Performance, Verbal, and Full-Scale raw scores, respectively. With covariate adjustment, water As remained significantly negatively associated with both performance and Full-Scale raw scores, explaining an incremental 4.33, 0.89, and 3.88% of the variance in Performance, Verbal, and Full-Scale raw scores, respectively. Results were similar when “IQ” outcomes were substituted for raw scores (data not shown).

### Dose–response relationships between water As and intellectual function.

**Figure 1:**
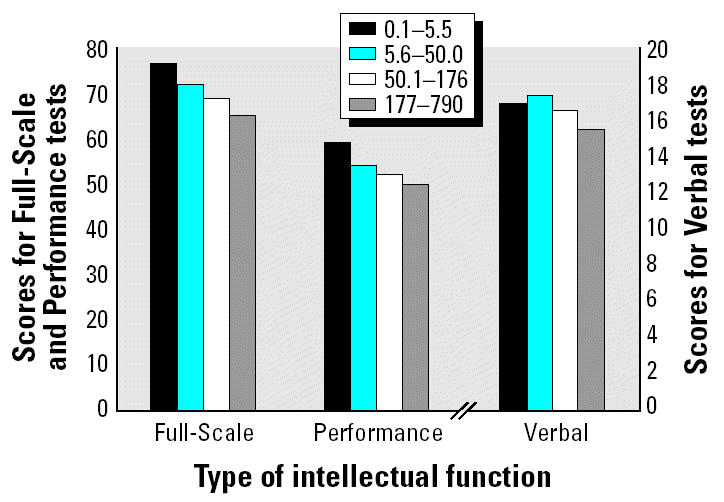
Adjusted scores by quartiles of water As for Full-Scale, Performance, and Verbal raw scores. In each case, adjustments were made for maternal education and intelligence, type of housing, child height and head circumference, and access to television.

**Figure 2:**
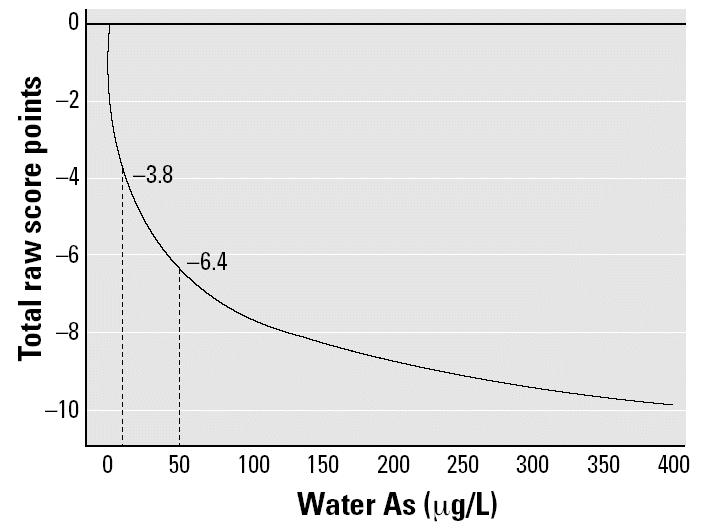
Continuous relationship between water As and Full-Scale raw score, adjusted as in Figure 1. The dotted perpendicular lines illustrate the loss in Full-Scale raw score associated with water As concentrations of 10 and 50 μg/L.

Figure 1 illustrates the adjusted Full-Scale, Performance, and Verbal raw scores by As quartile. As water As increased, there were dose-dependent changes in adjusted and unadjusted (data not shown) scores. With adjustment, compared with the lowest quartile of As exposure, the third and fourth quartiles had significantly lower scores on both Full-Scale (*B* = −7.8 and −11.3, *p* < 0.05 and *p* < 0.01, respectively) and Performance raw scores (*B* = −7.3 and −9.7, *p* < 0.05 and *p* < 0.01, respectively). The highest exposure quartile was marginally lower on Verbal raw score than the lowest exposure quartile (*B* = −1.6, *p* < 0.10). The relationship between water As (measured continuously) and Full-Scale raw score is illustrated in Figure 2. Water As concentrations of 10 and 50 μg/L were associated with decrements in Full-Scale raw scores of 3.8 and 6.4 points, respectively.

### Relationship between urinary As, As metabolites, and intellectual function.

We examined relationships between total urinary As concentration, as micrograms per gram creatinine, and child intellectual function. After adjustment for core variables, the associations between urinary As and measures of intellectual function were not statistically significant for Full-Scale (*B* = −2.9, *p* = 0.09), Performance (*B* = −2.2, *p* = 0.14), or Verbal scores (*B* = −0.7, *p* = 0.11) but were in the anticipated direction.

**Figure 3:**
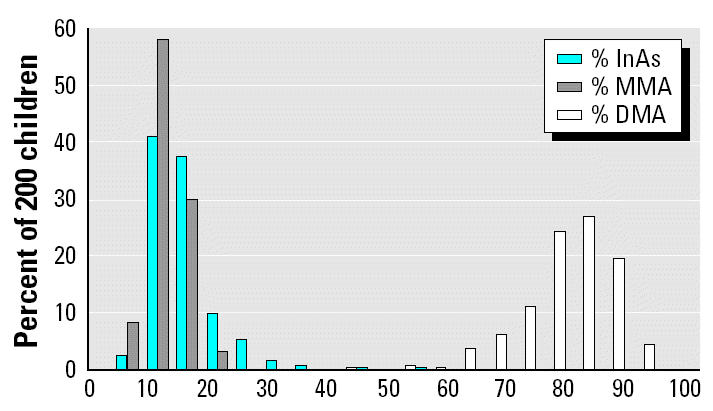
Frequency distributions of InAs, MMA, and DMA in urine, expressed as a percentage of total urinary As.

Urine samples were analyzed by high-performance liquid chromatography/ICP-MS for the relative amounts of InAs, MMA, and DMA. The percentages of InAs, MMA, and DMA were calculated after subtracting the contribution of AsC and AsB to total As concentration. The mean ± SD AsC and AsB concentrations were 3.9 ± 3.5 μg/g creatinine and 5.0 ± 7.5 μg/g creatinine, respectively. The frequency distributions of InAs, MMA, and DMA are illustrated in Figure 3. There was a wide variability in the extent to which children eliminated As in the dimethylated form. On average, the percentages of urinary As eliminated as InAs, MMA, and DMA were 12.2, 8.9, and 74.1%, respectively. We posited that children who were poor methylators might be particularly adversely affected by As. However, when both DMA and urinary As were included in the core model, DMA failed to make a significant contribution to intellectual function and did not alter the estimates for total urinary As.

### Relationships between BPb, Hgb, and intellectual function.

Analyses predicting intellectual raw scores from other hematologic measures, adjusted for the same demographic features, were conducted for the subset of 107 children providing blood samples. No significant associations were detected for log BPb or for Hgb on Verbal, Performance, or Full-Scale raw scores or “IQ,” with or without the inclusion of water As (data not shown).

## Discussion

This is the first systematic study of effects of As on children’s intellectual function. Exposure to As from drinking water was associated with reduced scores on measures of intellectual function, before and after adjusting for sociodemographic features known to contribute to intellectual function. With covariate adjustment, water As remained significantly negatively associated with both Performance and Full-Scale raw scores. Exposure to As was associated with reduced intellectual function, in a dose–response manner, such that children with exposures > 50 μg/L received significantly lower Performance and Full-Scale scores than did children with exposures < 5.5 μg/L. The association was stronger for well-water As than for urinary As. Children in the highest quartile of water As scored approximately 10 points lower in Performance raw scores than did those in the lowest quartile.

We have made diligent efforts to reduce the consumption of As-contaminated water in the Araihazar population since our original well survey was conducted in the first half of 2000. For example, each well was labeled to indicate As concentrations > and < 50 μg/L, with either a skull and cross-bones or a picture of a child drinking water. In addition, a village education program that encouraged well switching (van Geen et al. 2002) successfully reached roughly half of all residents. Beyond these, new low-As private and community wells have been installed in parts of the region during this time frame (van Geen et al. 2003a). It is therefore likely that some recent reduction in these children’s As exposure occurred between January 2001 (when well labeling began) and the summer of 2002 (when children were assessed). Indeed, in our simultaneous prospective cohort study in adults, repeated measurements of urine As concentrations over the same interval have declined. Because urinary As reflects recent exposure, reduced exposure may explain the weaker associations between intellectual function and levels of As in urine, compared with levels in water.

Two published studies of As exposure also found adverse associations with children’s intellectual function. In a small (*n* = 80) sample of children from a Pb smelter area in Mexico, Calderon et al. (2001) found negative associations between children’s urinary As and Verbal intelligence, controlling for a small set of demographic factors. Although investigating anthropogenic exposure to As and Pb, that study did not consider other potential toxicants to which nearby residents were exposed. In a second ecologic study, Tsai et al. (2003) compared adolescents in Taiwan from regions with and without elevated As in well water, with no measure of individual exposure. With minimal control for sociodemographic factors, adolescents in the exposed group showed inconsistently poorer scores on Performance-type tests; some outcomes were adversely affected in adolescents with low exposure (but not in those with high exposure) relative to those without exposure.

### As metabolism.

Humans excrete MMA and DMA after ingestion of arsenate or arsenite, but the extent of metabolism is remarkably variable and may influence both pre- and postnatal toxicity (Hopenhayn-Rich et al. 1996). This variability in methylation is likely due to both genetic (Chung et al. 2002; Vahter et al. 1995) and dietary factors. Maintenance of an adequate supply of the ultimate methyl donor (i.e., *S*-adenosylmethionine) requires an adequate supply of dietary folate and B vitamins. Two previous studies of As metabolism in very small groups of children have suggested that children are poor methylators compared with adults (Chowdhury et al. 2003; Concha et al. 1998). For example, children in two exposed villages in Argentina eliminated 49 and 42%, respectively, as InAs in urine, significantly more than did women in the same villages (25 and 29%, respectively) (Concha et al. 1998). In our study, children were not poor metabolizers. Only 12.2% of urinary As was in the InAs form; mean levels of MMA and DMA were 8.8 and 74.1%, respectively. These metabolite levels compare favorably with those reported for adults (Concha et al. 1998; Hopenhayn-Rich et al. 1996) in various parts of the world and with those of a subset of 300 adults in our cohort study (data not shown).

### Mn and Pb exposure.

The relationship between water Mn and children’s intellectual function suggested a possible adverse effect, above and beyond the contribution of social factors. However, that relationship did not persist once water As was added to the regression model. This study was not designed to examine the effects of Mn exposure on intellectual function, and in fact there was a moderate and significant positive association between water Mn and water As. A rigorous examination of the possible relationship between Mn exposure and intellectual function in children calls for a study design in which As exposure is extremely low.

We did not observe the anticipated relationship between BPb and child intellectual function. Our ability to detect this relationship was severely hampered by low statistical power, because approximately half of the study children refused to provide a blood sample.

### Limitations.

We cannot comfortably make a statement about IQ points lost in relationship to As exposure, because of limitations in the application of the U.S. standardization norms to the generation of IQ scores in the present study population. As we have pointed out, the lack of measures of intelligence standardized for use in Bangladesh hampers our ability to draw inferences about IQ points lost at given levels of exposure. Although we have followed sound procedures (derived from our related work in Kosovo) for adapting a widely used instrument to this very different cultural setting, and although we have avoided, for the most part, drawing conclusions about IQ, the measures used here are not measures of IQ, and the absence of standardized measures remains a limitation.

Employing raw scores avoids many pitfalls that would result from using nonstandardized procedures; however, the removal of culturally bound items and subscales diverges from common practice. On the other hand, other simpler predictors of child intellectual function, such as maternal education and child height and head circumference, were significantly related to intellectual raw scores in the expected directions. This gives us confidence in the validity of the observed associations with As. To provide estimates of the impact of As exposure on IQ that would be more directly useful to policy makers, future research should either standardize an IQ test for Bangladesh (a considerable undertaking) or replicate the present effort in a well-defined sample of Western children. Given that the prevalence of malnutrition is quite high in Bangladesh, and that children in our study were of small stature relative to U.S. norms (although not anemic), the dose–response relationship in U.S. children may be different.

The present investigation examines a single age group at a single point in time. We do not know whether the present level of deficit can be detected earlier, whether continued exposure is associated with increased intellectual loss, or, conversely, whether a reduction in exposure would be associated with improved functioning. Better understanding of the exposure–outcome relationship could be obtained by following a group of children from an earlier age and tracking both exposure and outcome regularly.

We believe that our finding of a strong association between As exposure and intelligence is both important and tragic and adds urgency to the need for effective remediation in Bangladesh and other regions of South Asia where consumption of As-contaminated water is prevalent. The global community has been slow in responding to the public health significance of As exposure in Bangladesh, despite the enormous scope of the problem. We hope that the present findings add a new sense of urgency to efforts aimed at alleviating and eliminating As exposure in Bangladesh.

## Figures and Tables

**Table 1:** Sample characteristics [no. (%) or mean ± SD].

Variable	No. (%)
Male	98 (48.8)
Television access	70 (34.8)
House type	
Thatched roof or poorer	20 (10.0)
Corrugated tin	149 (74.1)
Concrete construction	32 (15.2)
Father’s occupation	
Other/missing	23 (11.4)
Laborer/farmer	47 (23.4)
Factory/other paid job	67 (33.3)
Business	64 (31.8)
Child age	10.0 ± 0.4*^a^*
Full-Scale “IQ”	53.0 ± 6.3
Verbal “IQ”	55.4 ± 5.2
Performance “IQ”	58.4 ± 8.0
Full-Scale raw score	70.5 ± 20.8
Verbal raw score	16.5 ± 5.1
Performance raw score	54.0 ± 17.4
Height (cm)	125.6 ± 6.5
Weight (kg)	21.9 ± 3.3
Body mass index (kg/m^2^)	13.8 ± 1.1
Head circumference (cm)	49.5 ± 1.4
Mother’s education (years)	2.9 ± 3.4
Father’s education (years)	3.7 ± 3.7
Mother’s age (years)	32.6 ± 6.7
Mother’s Raven’s score	14.4 ± 3.5
Water Mn (μg/L)	1,386 ± 927
Water As (μg/L)	117.8 ± 145.2
Urinary As (μg/L)	116.6 ± 148.8
Urinary creatinine (mg/dL)	43.3 ± 34.1
Urinary As (μg/g creatinine)	296.6 ± 277.2
Hgb (g/dL)	12.6 ± 1.1
BPb (μg/dL)	10.1 ± 3.3

Except where noted, sample size is 201. Values are no. (%) except where indicated.

a Mean ± SD.

**Table 2:** Prediction of Verbal, Performance, and Full-Scale raw scores from water As before and after covariate adjustment.

Variable	Performance	Verbal	Full-Scale
Before adjustment			
Water As	−1.84*^#^*	−0.32*	−2.16*^#^*
After adjustment			
Maternal education			
None	−6.25*^##^*	−2.57*	−8.82*
1–5 years	−2.75	−0.98	−3.74
5–13 years	(Reference group)			
Maternal intelligence	0.46	−0.02	0.44
House type			
Thatched roof or poorer	−7.03	−1.93	−8.96*^##^*
Corrugated tin	−1.50	−0.34	−1.83
Concrete	(Reference group)			
Television access	3.53	1.60*	5.13*^##^*
Height	0.69*^#^*	0.13*	0.81*^#^*
Head circumference	2.31**	0.78**	3.08**
Water As	−1.45**	−0.19	−1.64**
Total *R*^2^ (%)	29.02	22.83	31.63

* *p* < 0.05,

** *p* < 0.01,

# *p* < 0.001,

## *p* < 0.10.
